# A Multifaceted Action of Phytochrome B in Plant Environmental Adaptation

**DOI:** 10.3389/fpls.2021.659712

**Published:** 2021-06-22

**Authors:** Jae Young Kim, June-Hee Lee, Chung-Mo Park

**Affiliations:** ^1^Department of Chemistry, Seoul National University, Seoul, South Korea; ^2^Plant Genomics and Breeding Institute, Seoul National University, Seoul, South Korea

**Keywords:** phyB, light, thermal sensing, drought, defense, environmental adaptation

## Abstract

Light acts as a vital external cue that conveys surrounding information into plant growth and performance to facilitate plants to coordinate with changing environmental conditions. Upon exposure to light illumination, plants trigger a burst of molecular and physiological signaling cascades that induces not only photomorphogenic responses but also diverse adaptive behaviors. Notably, light responses and photomorphogenic traits are often associated with plant responses to other environmental cues, such as heat, cold, drought, and herbivore and pathogen attack. Growing evidence in recent years demonstrate that the red/far-red light-absorbing phytochrome (phy) photoreceptors, in particular phyB, play an essential role in plant adaptation responses to abiotic and biotic tensions by serving as a key mediator of information flow. It is also remarkable that phyB mediates the plant priming responses to numerous environmental challenges. In this minireview, we highlight recent advances on the multifaceted role of phyB during plant environmental adaptation. We also discuss the biological relevance and efficiency of the phy-mediated adaptive behaviors in potentially reducing fitness costs under unfavorable environments.

## Introduction

Light acts as a central environmental cue that triggers a variety of adaptive behaviors in plant organs, termed photomorphogenesis (Arsovski et al., 2012). Plants have evolved multiple photoreceptors, each having distinct photochemical properties, that sense changes in light intensity, direction, wavelength, and diel and seasonal rhythmicity. Among them, the red (R)/far-red (FR) light-absorbing phytochrome (phy) photoreceptors have been most extensively characterized (Pham et al., 2018).

While light is critical for plant growth and survival, high light intensity often accompanies temperature increases over a physiological range in nature (Wang and Dickinson, 2013), which provoke life-threatening constraints, such as disturbance of physiological performance and excessive evaporation of soil water (Monroe et al., 2018), leading to drought stress and elevation of salt concentrations in the soil (Uno et al., 2000). On the other hand, during the winter season, low light intensity and alterations in light quality often accompany temperature drops, which impose cold shock on plants (Szeicz, 1974; Wang and Dickinson, 2013). The diel and seasonal rhythmic cycles of light and temperature also profoundly influence the behavior patterns of herbivores and pathogens (Koelle et al., 2005; Abarca, 2019). It is now perceived that light is intimately associated with plant responses to abiotic and biotic factors by sharing signaling molecules (Huot et al., 2014). The best characterized are the signaling networks that mediate plant responses to light and temperature. The phyB photoreceptor acts as a thermosensor during plant thermomorphogenesis, a distinct array of morphogenic and architectural reshaping events occurring under warm ambient temperatures (Casal and Balasubramanian, 2019).

Accumulating evidence indicate that phyB also plays a role in maintaining plant fitness under abiotic and biotic stress conditions (Gonzalez et al., 2012; De Wit et al., 2013), which are also physiologically interconnected with light and temperature responses. Therefore, unraveling the roles of phyB as a signaling integrator of multiple surrounding information would facilitate our understanding on how plants strive to adapt to the co-occurring environmental changes and contribute to exploring ways of developing climate-smart crops having an improved adaptive capacity to environmental stressors.

The primary task of this minireview is to summarize recent advances on the versatile roles of the phy photoreceptors, with focus on phyB, during thermomorphogenesis and adaptive responses to abiotic and biotic challenges and discuss a unified phyB-mediated signaling network that integrates multiple signaling cues into plant growth and adaptive morphogenesis. Considering that plant stress adaptation is tightly linked with managing fitness costs (Karasov et al., 2017), we also discuss how plants maintain metabolic balance by modulating intrinsic energy balance and minimizing energy costs.

## The Phytochromes in Photomorphogenesis and Beyond

**FIGURE 1:**
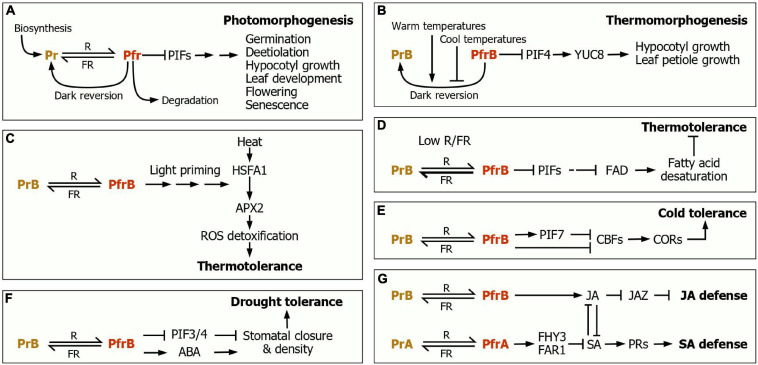
Phytochrome control of plant morphogenic and adaptive responses. **(A)** Photomorphogenesis. The dynamic interconversion between the Pr and Pfr spectral forms, depending on light wavelengths, serves as a molecular switch in regulating a variety of morphogenic and developmental events, covering from seed germination to flowering and senescence. In the dark, the Pfr form is steadily converted to the Pr form, termed dark reversion. The phytochrome function is also regulated at the steps of its biosynthesis and degradation. **(B)** Thermomorphogenesis. The PfrB-to-PrB dark reversion is accelerated at warm temperatures but delayed at cool temperatures. The thermomorphogenesis promoter PIF4 activates the expression of the *YUC8* auxin biosynthetic gene, leading to the promotion of hypocotyl and leaf petiole growth. **(C)** Light priming of thermotolerance response. The PfrB signals prime the induction of the *APX2* gene, whose gene product detoxifies ROS, leading to the induction of thermotolerance. **(D)** Enhancement of thermotolerance under plant canopy. Under this severe shade condition, low R/FR shifts the phyB photoconversion toward PrB, and thus the *FAD* expression is repressed, leading to the reduction of fatty acid desaturation and the resultant enhancement of thermotolerance. **(E)** Light regulation of cold tolerance. The CBF-COR regulon plays a key role during cold acclimation. Under high R/FR, the PfrB form, in conjunction with PIF7, suppresses the expression of the *CBF* genes, leading to the reduction of cold tolerance. In contrast, under low R/FR, as frequently encountered during twilight in the fall season, the phyB photoconversion is shifted toward Pr, leading to the activation of the CBF-COR regulon. **(F)** The phyB-mediated regulation of drought tolerance. The PfrB form increases stomatal density and induces stomatal closure. Under drought conditions, where ABA biosynthesis is elevated, the PfrB signals increase ABA sensitivity/responsiveness. These signaling cascades cause the enhancement of drought tolerance. **(G)** The phytochrome control of defense responses. The phytochrome-mediated light signals regulate SA biosynthesis and perception to induce the expression of *PR* genes during SA responses. Under shade conditions, JA-mediated defense responses are attenuated via a JAZ-mediated transcriptional control. The JA and SA responses are linked through signaling crosstalks.

Plants possess several distinct groups of photoreceptors, such as phy photoreceptors, cryptochromes, phototropins, and UV-sensing photoreceptors (Casal, 2000). LOV domain-containing F-box proteins, such as ZEITLUPE, FLAVIN-BINDING, KELCH REPEAT, F-BOX 1, and LOV KELCH PROTEIN 2, have been shown to act as blue light photoreceptors (Ito et al., 2012). In addition, neochrome photoreceptors have been identified in ferns and algae (Suetsugu et al., 2005). There are five phy members, phyA to phyE, in *Arabidopsis*, which mediate both distinct and overlapping photomorphogenic traits (Pham et al., 2018). PhyB is a light-stable phy photoreceptor, and its roles have been explored in a variety of photomorphogenic processes (Figure 1A; Rausenberger et al., 2010; Pham et al., 2018). Interestingly, recent studies have shown that phyB-mediated light responses are widespread in all plant organs and tissues (Ha et al., 2018; Oh et al., 2018). While the shoot phyB mediates photomorphogenic shoot growth (Pham et al., 2018), the root phyB is photoactivated by shoot-driven light signals or stem-piped light and regulates growth and gravitropic behaviors in the roots (Lee et al., 2016, 2017). Furthermore, the phyB-mediated light signals are efficiently transmitted across different plant tissues, contributing to synchronized plant growth (Ha et al., 2018).

The critical component of the phy actions is the dynamic photoconversion between the R light-absorbing Pr and the FR light-absorbing Pfr forms. The Pfr form is considered to be a physiologically active form in most light responses (Klose et al., 2015). The Pr-Pfr photoconversion initiates diverse light signaling events by modulating either its interactions with downstream signaling mediators, such as PHYTOCHROME-INTERACTING 4 (PIF4), its nucleocytoplasmic distribution, or by modulating its nuclear speckle formation (Sakamoto and Nagatani, 1996; Huang et al., 2019). The photoconversion is also associated with the chemical modifications of the phy proteins and their protein stability (Klose et al., 2015). In addition, the phy photoreceptors are also responsive to light intensity, direction, and diel and seasonal rhythmicity (Park et al., 2018). It is particularly interesting that the Pr-Pfr photoconversion is triggered by not only light wavelengths but also temperature changes. It is known that the Pfr-to-Pr dark reversion is accelerated at warm temperatures but suppressed at low temperatures (Jung et al., 2016; Legris et al., 2016).

## Thermochemical Control of phyB Function During Thermomorphogenesis

Along with light signals, temperature cues are one of the most important environmental factors that profoundly affect plant growth and architecture. Plants are capable of actively responding to cold and warm average temperatures and rhythmic thermal fluctuations via an array of diverse morphological and developmental reshaping events, collectively termed thermomorphogenesis (Casal and Balasubramanian, 2019; Vu et al., 2019). In particular, subtle changes within a physiological warm temperature range induce distinct thermomorphogenic processes, such as increase of leaf hyponasty, acceleration of hypocotyl and petiole growth, and formation of small thin leaves, which are widely studied in view of their climatic relationship with global warming in recent years (Koini et al., 2009; Park et al., 2019). It has been proposed that the warm temperature-induced plant thermomorphogenic traits ensure plant survival and optimal growth by protecting the light-labile shoot apical meristems from the hot soil surface and accelerating evaporative leaf cooling (Crawford et al., 2012).

A most exciting observation is that ambient temperatures influence the Pfr-to-Pr dark reversion (Jung et al., 2016; Legris et al., 2016). At warm temperatures, the Pfr-to-Pr dark reversion is accelerated, thus also termed thermal reversion (Legris et al., 2016). The accelerated Pfr-to-Pr reversion leads to the transcriptional upregulation of growth-related downstream genes via PIF4, such as *YUC8* encoding an auxin biosynthetic enzyme (Figure 1B; Pham et al., 2018). It is likely that the dual role of phyB as photoreceptor and thermosensor via the Pfr-Pr conversion contributes to coordinately integrating light and temperature information into growth and morphology. For detailed signaling mechanisms and regulatory schemes underlying plant thermomorphogenesis, the readers refer to recent excellent reviews (Casal and Balasubramanian, 2019; Vu et al., 2019).

## phyB Function During Plant Adaptation to Temperature Extremes

### Thermotolerance

Temperature increases beyond a physiological range impose harmful effects on plant growth, development, and survival (Hasanuzzaman et al., 2013). Therefore, plants have evolved versatile thermoadaptive mechanisms to cope with heat shock (Ohama et al., 2017). In nature, especially in temperate regions, high temperatures occur mostly in accordance with the light-dark cycles, and thus it is easily imaginable that light signaling is linked with high temperature responses. Indeed, recent studies reveal that phyB plays an important role in the photoregulation of heat acclimation (Arico et al., 2019; Han et al., 2019).

A recent study has shown that light pretreatment, called light priming, greatly improve thermotolerance and phyB plays a key role in the light priming of thermotolerance responses (Figure 1C; Han et al., 2019). At high temperatures, reactive oxygen species (ROS) accumulate in plant cells, inducing oxidative damages on biomolecules (Pospíšil, 2016). The light priming activates the phyB function, and phyB stimulates the expression of *APX2* gene, encoding a ROS scavenging enzyme, in a PIF-independent manner, enhancing thermotolerance.

Meanwhile, it has been reported that plant thermotolerance varies greatly in response to R/FR ratio (Arico et al., 2019). Seedlings grown under low R/FR ratio exhibit an enhanced thermotolerance compared to those grown under high R/FR ratio, and the light wavelength-dependence of thermotolerance requires both phyB and PIF transcription factors (Figure 1D). Under low R/FR ratio, as frequently encountered under plant canopy, the PIF activity is induced, resulting in the suppression of the *FATTY ACID DESATURASE* (*FAD*) gene encoding a fatty acid desaturase (Arico et al., 2019). At high temperatures, ROS selectively destabilize unsaturated fatty acids, reducing thermotolerance (Anjum et al., 2016). Therefore, under shade, the reduction of FAD production is associated with an enhancement of thermotolerance. Overall, it is evident that the photochemical balance between the Pr and Pfr forms constitutes a critical signaling element governing thermotolerance responses.

It is interesting that two phyB-related light quality conditions, light priming and R/FR ratios, regulate thermotolerance in opposite ways via distinct signaling pathways (Arico et al., 2019; Han et al., 2019). This paradox could be explained by considering natural light environments. In nature, plants are often exposed to shade caused by neighbor plants, which accompanies low R/FR conditions. While phyB would be shifted dominantly toward the Pr form under such light conditions, temperature would be still high, and thus enhancement of thermotolerance would be beneficial to plant survival. However, during the night, temperatures would not be high, and the induction of thermotolerance is not necessarily required.

### Cold Tolerance

Cold temperatures adversely affect plant growth and development, eventually causing plant death. Molecular genetic studies have identified a large group of functional components involved in cold temperature perception and signaling (Guo et al., 2018). The best characterized is the CBF-COLD*-*COR regulon, in which the CBF transcription factors rapidly induce the expression *COR* genes, whose gene products include enzymes involved in the biosynthesis of osmoprotectects, lipid metabolism, cell wall modifications, and protein kinases that mediate growth hormone signaling (Zhao et al., 2016; Shi et al., 2018).

Cold shock is often associated with the light-dark cycles during diel or seasonal transitions, necessitating that light signaling is linked with cold temperature responses (Maibam et al., 2013). Indeed, growing evidence indicate that light responses and cold acclimation share many signaling mediators and regulatory mechanisms. It has been shown that changing photoperiods influence the development of cold tolerance in *Arabidopsis* (Lee and Thomashow, 2012). Plants grown under short days (SDs) exhibit a greater cold tolerance compared to what observed in long day (LD)-grown plants. Under LDs, the expression of *PIF4* and *PIF7* gene is upregulated. The PIF transcription factors suppress the expression of *CBF* genes. Moreover, the cold tolerance phenotype and the *CBF* expression are not discernibly changed regardless of daylengths in phyB-deficient mutants (Lee and Thomashow, 2012), indicating that phyB is required for the photoperiod-mediated control of cold tolerance.

In support of the importance of phyB in cold tolerance, R/FR light ratio is crucial for cold tolerance responses (Franklin and Whitelam, 2007; Wang F. et al., 2016). Tomato plants grown under high R/FR ratio exhibit a reduced cold tolerance compared to those grown under low R/FR ratio in a phyB-dependent manner (Wang F. et al., 2016). In *Arabidopsis*, the photoactivated phyB promotes the transcriptional activity of PIF7, which suppresses *CBF* gene expression (Figure 1E; Franklin and Whitelam, 2007; Kidokoro et al., 2009). These observations indicate that the interconversion between the phyB Pr and Pfr forms serves as a molecular switch that integrates light signals into the development of cold tolerance.

## phyB-mediated Enhancement of Drought Tolerance

Drought imposes detrimental effects on vegetative growth, metabolism, and reproductive performance. The symptoms of drought stress are featured by stomatal closure and reduction of cell growth. It occurs when plants experience low rainfall, high salinity, temperature extremes, and high light intensity in nature (Farooq et al., 2012).

The intimate linkage between excessive light exposure and drought stress is readily recognized in nature. While high light irradiance and sufficient soil water content sustain photosynthesis, excessive high light irradiance accelerates water evaporation from plant body and the soil, causing water deficit stress (Farooq et al., 2012). Especially, during the summer season in temperate regions, high temperature-driven drought conditions are prominent during the day (Monroe et al., 2018). Considering the simultaneous occurrence of high temperature, strong solar irradiance, and drought, it is anticipated that plant responses to drought would be associated with light signaling.

The abscisic acid (ABA) phytohormone regulates stomatal movements and drought-responsive gene expression. Under drought conditions, ABA accumulates mainly in the leaf vascular tissues, from which it spreads to virtually all plant tissues to enhance stomatal density and closure (Bright et al., 2006; Tanaka et al., 2013). Recent studies illustrate that phyB-mediated light signals differentially modulate water preservation in plant body. Under well-watered environments, phyB promotes stomatal opening and development, possibly via COP1 or PIFs, to obtain carbohydrates as much as possible (Figure 1F; Wang et al., 2010). However, when the availability of soil water is limited under high light irradiance, the phyB Pfr form stimulates ABA sensitivity, leading to reduced stomatal opening (Gonzalez et al., 2012). It is also known that PIF overexpression promotes stomata closure, leading to drought tolerance in crop plants (Gao et al., 2018). The molecular linkage of drought responses with light signaling is likely to provide an adaptive strategy, by which plants foresee upcoming water deficit conditions under high light irradiance.

## Phytochrome Control of Plant Defense

Insect herbivores and pathogens impose a destructive threat on plant survival and propagation. Plants therefore have developed versatile defense mechanisms to cope with these biotic stressors, and a large collection of pathogen- and herbivore-inducible genes and signaling molecules have been identified in various plant species (Dodds and Rathjen, 2010), among which two stress phytohormones, salicylic acid (SA) and jasmonic acid (JA), have been most extensively studied.

It is notable that the induction pattern and altitude of plant defense responses are widely affected by light and temperature. The coordinated linkage between plant defense and light cycles has been extensively studied (Genoud et al., 2002; Wang W. et al., 2016). The onset and progression of defensive behaviors is reshaped by light intensity and wavelength and light-dark transitions (Griebel and Zeier, 2008; De Wit et al., 2013). The light regulation of plant defense is illustrated in two major aspects. It is known that the behavioral, and physiological patterns of herbivore and pathogen are synchronized with the diel cycles of light and thus plants are capable of predicting the occurrence of their attacks (Koelle et al., 2005; Abarca, 2019). Another factor to be considered is growth-defense tradeoffs that plants have to deal with. While defense responses are costly, requiring metabolic resources and energy, plants have limited resources. Therefore, excessive allocation of metabolite and energy to defense responses would cause growth reduction (Huot et al., 2014). It is anticipated that plants have to precisely monitor surrounding light information to balance fitness benefits and costs (Huot et al., 2014).

As predicted from the synchronization of herbivore and pathogen attack with light cycles and the growth-defense tradeoffs, recent studies have shown that light signals play an important role in modulating defense responses (Figure 1G; Griebel and Zeier, 2008; Wang W. et al., 2016). Under low R/FR conditions, JA contents are reduced, and plants exhibit a low sensitivity to SA or JA treatments in inducing defense responses (De Wit et al., 2013). Accordingly, the phyB-deficient mutants having a constitutive shade avoidance phenotype exhibit a hypersusceptibility to pathogens (De Wit et al., 2013). Meanwhile, under dark conditions, SA biosynthesis and sensitivity of plants are reduced, and defense responses are compromised (Genoud et al., 2002; Griebel and Zeier, 2008). The *phyA phyB* double mutants also exhibit a significant reduction in SA sensitivity and systemic acquired resistance, indicating that phy-mediated light signaling is essential for SA-associated defense responses. In contrast, under high R/FR conditions, the light stabilization of FHY3 and FAR1, two homologous transcription factors that are important for phyA signaling (Wang and Deng, 2002), negatively regulate SA biosynthesis and its signaling (Wang W. et al., 2016). It is likely that the phyA-mediated light regulation of SA biosynthesis and signaling contribute to finetuning SA-associated defense responses (Figure 1G). It is therefore obvious that phyB plays a key role in enhancing both SA and JA-mediated defense responses.

Moreover, it has been demonstrated that phyB regulates JA biosynthesis and sensitivity, depending on R/FR ratio (Cerrudo et al., 2017; Fernandez-Milmanda et al., 2020). Under low R/FR conditions, the expression of *SULFOTRANSFERASE 2A* (*ST2A*) gene, which functions in JA metabolism, is elevated in a phyB/PIF-dependent manner (Fernandez-Milmanda et al., 2020). The *ST2A* induction is linked with the reduction of JA accumulation, weakening JA responses. Meanwhile, low R/FR ratio and *phyB* mutation induce rapid degradation of DELLA proteins (Leone et al., 2014), well-known repressors of JAZ function (Hou et al., 2010), to increase JAZ10 protein stability, resulting in a reduced JA sensitivity. It is obvious that phyB and phyA are crucial for the light reinforcement of defense responses.

## The P_*R*_-P_*FR*_ Photoconversion as a Core Regulator of Plant Adaptation

**FIGURE 2:**
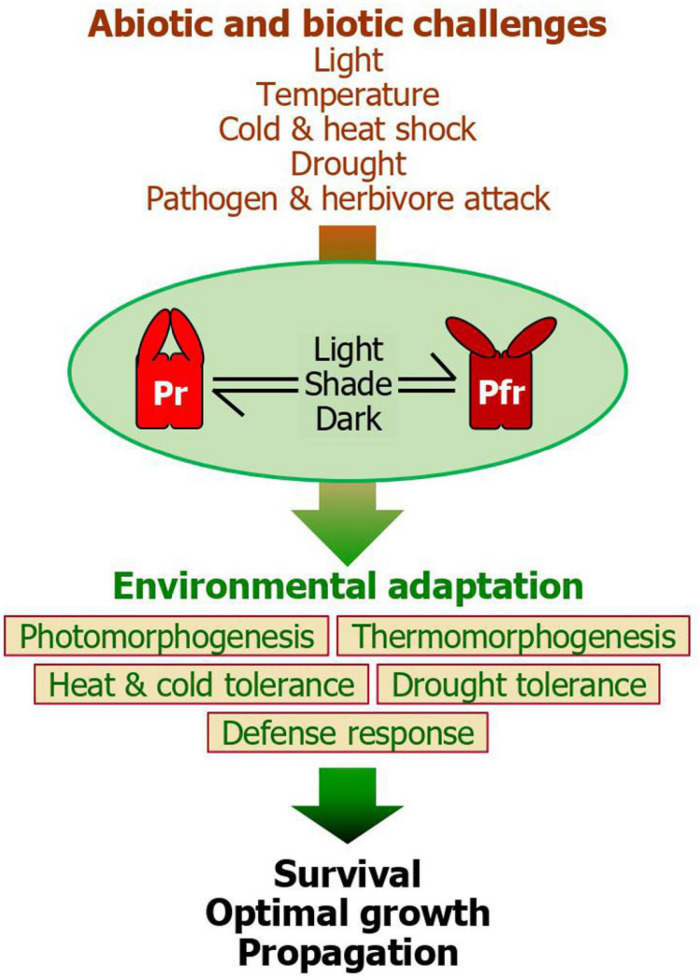
A unified model for the phytochrome function during plant environmental adaptation. While the Pr-Pfr photoconversion plays a central role during photomorphogenic responses, it also integrates a range of light information into plant adaptive behaviors in response to various abiotic and biotic challenges, such as varying light environments, ambient temperatures, heat and cold shocks, drought, and herbivore and pathogen attacks. Among the five phytochrome members in Arabidopsis, phyB plays a major role in these adaptive signaling networks. It is known that the red light-stimulated accumulation of the Pfr form activates plant adaptation to heat, drought, and biotic attacks in most cases. On the other hand, the accelerated conversion of the phytochromes to the Pr form, as frequently occurring during diel and seasonal transitions in temperate regions or under plant canopy, helps plants to overcome cold shock. Through the phytochrome-mediated adaptation behaviors, plants are able to ensure survival and sustain optimal growth and propagation, enhancing plant fitness. The intimate linkage between photomorphogenesis, which culminates in an optimization of photosynthesis, and adaptive behaviors would contribute to minimizing fitness costs and enhancing plant performance under fluctuating environments.

Molecular genetic studies and genome-scale approaches in model and crop plants indicate that they have evolved diverse mechanisms and signaling networks for efficient sensing and transducing surrounding signals into morphological, physiological, and biochemical processes, culminating in environmental adaptation and survival (Arsovski et al., 2012). The best characterized is plant responses to light information and associated morphogenic responses. Plant photomorphogenesis is governed by multiple photoreceptors, among which the phy photoreceptors are best characterized (Casal, 2000; Pham et al., 2018). Interestingly, plant photomorphogenesis is closely associated with plant responses to environmental stimuli, including both abiotic and biotic factors (Arsovski et al., 2012). As a result, plants are capable of orchestrating their growth and adaptive behaviors with surrounding growth conditions. For example, it is believed that the PfrB is considered to reinforce adaptive behaviors in response to light-related abiotic stress conditions, such as heat, drought, and biotic challenges (Gonzalez et al., 2012; De Wit et al., 2013; Han et al., 2019). In contrast, under low R/FR conditions, as frequently observed during the winter seasons, the diminution of PfrB triggers the derepression of cold response genes, facilitating plants to overcome cold environments. Overall, it is evident that the Pr-Pfr photoconversion acts as a central integrator of various environmental cues in facilitating plant fitness (Figure 2).

The intimate linkage between light and other environmental cues reflects two distinct ecological consequences. One aspect is the signaling interactions of light with other cues. In nature, light signals oscillate in accordance with other environmental cues, such as temperature and drought (Wang and Dickinson, 2013; Monroe et al., 2018). Therefore, the behavior patterns of insect herbivore and pathogens are synchronized with light cycles in many cases. In this regard, it is believed that light regulation of plant morphogenic and adaptive behaviors provides a way of harmonizing its growth and propagation with changing environments. Another aspect is related with the growth-defense tradeoff concept. Plant photomorphogenesis reshapes body architecture to optimize growth and energy gain (Arsovski et al., 2012). The management of photosynthetic capacity during diel and seasonal cycles enhances plant fitness to changing environments (Athanasiou et al., 2010). Thus, it is necessary that plants pay fitness costs for optimizing carbohydrate allocation to balance energy gain and consumption. Overcoming unfavorable conditions, which demand prioritization toward survival rather than growth, are facilitated by paying fitness costs that include energy shift toward survival and adaptation.

## Conclusion and Perspectives

We summarized recent findings on the multifaceted role of phyB in plant environmental adaptation and discussed its potential role in maintaining plant fitness under fluctuating environments. In natural habitats, multiple environmental changes occur in a complex manner. While the patterns of temperature and drought cycles and the propagation patterns of herbivore and pathogen cooscillate in a regular manner with light cycles in many cases, others, such as temperature extremes, occur somewhat independent of light cycles. Nevertheless, light signaling is closely associated with most of abiotic and biotic signals by sharing some signaling molecular and mechanisms. It is believed that the phyB-mediated signaling networks help plants to manage fitness costs in adapting to environmental alterations. Therefore, signaling interactions and managing the growth-defense tradeoffs would have to be considered for better improvements of adaptive traits.

## Author Contributions

C-MP and JYK designed the concept and organization of the manuscript. C-MP and JYK wrote the manuscript with helps of J-HL. All authors contributed to the article and approved the submitted version.

## Conflict of Interest

The authors declare that the research was conducted in the absence of any commercial or financial relationships that could be construed as a potential conflict of interest.
